# College Clinical Service of Dr. Jones

**Published:** 1859-04

**Authors:** 


### ARTICLE III.

### *College Clinical Service of Dr. Jones.*

Jan. 12th.—You are aware that this case has been before you several times ; the patient is twelve years old. Miss M—— is considered as having a strumous diathesis, and is at this time suffering mainly from otirrhœa as a sequence of scarlatina; and this, let me assure you, is no unfrequent occurrence; it is always a troublesome and unpleasant affection; often painful, and in some instances, terminates fatally. Sometimes the disease subsides in the course of a few weeks or months; and in others it annoys the patient through life. Otitis or inflammation of the mucous membrane of the ear, is known to exist, by pain, more or less excruciating in the part; unpleasant humming in the ear; dull headache; giddiness; stupor, and the discharge of a puriform, or purulent liquid from the meatus auditorius externus, and of a very offensive character. In some cases this liquid makes its way through the eustachian tube into the fauces. It may be external, when the inflammation exists only in the external meatus, or internal when the cavity of the tympanum is involved; sometimes its ravages extend to the brain. It may, as already intimated, be acute or chronic—an example of the latter form, is exhibited by the case before you, and may be properly denominated consequential otirrhœa, or a puriform discharge from the external ear. The etiology, symptomatology, pathology and treatment of this case was thoroughly considered at a previous clinic, and the patient, as you have just heard, reports herself gradually improving, from the treatment then prescribed, and which consisted of the hydriodate of potassa, and vegetable tonics internally, warm clothing, nutritious diet, out-door exercise, and the tincture of iodine thrown into the ear—all of which is ordered to be continued. Long continued vesication immediately behind or below the ear, or even upon the nape of the neck, has been found serviceable in cases similar to this, and should be tried when the disease is obstinate. The auditory apparatus is occasionally so extensively diseased or destroyed, as to eventuate in almost total deafness.

Mr. L——, aged about forty-five years, you have been told, is laboring under valvular disease of the heart, and we fully concur in this diagnosis—his case has been minutely examined before, and explained to the class. Digitalis was prescribed, and he reports himself more comfortable now than prior to its use. For dyspeptic symptoms he was also directed to take the compound decoction of gentian and columbo—and a further continuation of this treatment is advised.

Mr. D——, about 40 years of age, from Middle Georgia, presents himself to us, and says, that ten years ago he had an affection of the kidneys, with edema of the legs, and from which he gradually recovered under the use of the mur. tinct. iron. The symptomatology of his case, at this time, does not clearly establish the diagnosis. By some, his disease is regarded as rheumatic in character—others apprehend the existence of morbus Brightii. You observe, that when he attempts to assume an erect position, in consequence of spasmodic distress, and actual pain in the epigastric region, he fails to do so; and that in making an effort to lie horizontally, the same set of symptoms are manifest—he can neither stand erect, nor lie down straight—some degree of flexion of the body is essential to an exemption from pain. Pressure upon the epigastrium produces little or no uneasiness; but when made upon the upper lumbar (at which point there is visible, some degree of tumefaction,) and lower dorsal vertebræ, much pain is excited, both at the point of pressure and about the precordia. This has been his condition in a greater or less degree, for the last eighteen months. You have just witnessed an analysis of his urine, by means of heat and nitric acid, and you have seen that it contains a considerable quantity of albumen; but this fact alone, or even in connection with other symptoms, does not satisfactorily settle the question of the true nature of his disease; it is but a link in the catenation of the semiology of his case; it does not reveal the true pathology of his disease, and it is obvious that it is difficult to diagnose it correctly. Has this man rheumatism of the diaphragm, or has he an affection of the renal glands, or is it either a functional or structural disease of the spinal marrow, or is he suffering mainly from what has been variously termed rhachialgitis, myelitis, or spinal irritation? The latter of which seems most probable.

Your text-book teaches you, that "it is a fact, which must always be borne in mind, in estimating the diseases of the cord, that it has a double office. *First*, that of receiving impressions from other parts of the body, and of transmitting influence to those parts which it may do independently of the brain. By the former office, it regulates conjointly with the ganglia, most of the organic functions, so far as nervous influence is concerned. By the latter, it is necessary to sensation and voluntary motion over the greater portion of the body. Two sets of phenomena, therefore, are consequent upon disease of the cord. In the first place, we have as the result of a perversion of its direct original action, symptoms of disorder in the digestive respiratory, circulatory, nutritive secretory and reproductive functions, all of which are more or less under the control of the spinal centres; and in the second place, those indicative of interrupted or deranged cerebral influence, such as perverted, deficient or abolished sensation, and in various degrees, the loss of the power of voluntary motion. Involuntary or spasmodic muscular contractions may arise either from disorder of the reflex spinal functions, seated in the centres, or from disease of the conducting filaments, hence, if we adopt this as our standard of semiological and diagnostic investigation, and duly consider the symptoms of the case before us, it is but rational to conclude, that it is spinal irritation, and for which a prescription will now be made:

Let Mr. D——— be scarified and cupped upon the tender portions of the spine twice weekly for one month, and then repeated vesications, with unguent canthar in the same localities, and the bowels be kept soluble with gentle aperients as calcined magnesia alone, or in combination with rhubarb. His diet should be of easy digestion, and moderately nutritious in character. Exercise on foot may be permitted to a limited extent. Should this course of treatment fail to benefit the patient, I would advise the use, successively, of the oil of turpentine, strychnia, or veratrine, in doses regulated by their effects upon the system.

### *Service of Dr. Powell.*

JAN. 10th.—*Prolapsus Uteri, with Hard Engorgement of the Cervix.*—Ann, a mulatto, aged forty-five, ordinary statue, rather thin, was robust and enjoyed good health, up to the birth of her first child, which took place in her sixteenth year. One year after, menstruation had been regularly established. Was very ill for three months after the birth of her child. Has been delicate ever since, but not sickly, and has never since been pregnant. She now complains of pain in the back; a feeling of numbness in the lower limbs, occasionally; sick stomach; frequent difficulty in urinating; habitual constipation, bowels rarely moved without the aid of medicine; daily headache, chiefly affecting the occiput; her tongue somewhat coated; pulse ninety; appetite poor. The Lecturer now made an examination by the touch, and stated that the uterus was slightly prolapsed, the os considerably dilated, and the cervix very much enlarged.

The speculum was introduced, which confirmed the opinion just expressed. The parts presented a normal appearance as to color, but the cervix was so enlarged that it presented more the appearance of the fundus than the neck.—The os was perfectly round and smooth.

Treatment.—In prolapsion of the womb, as in all other diseases, the cause should be first discovered and removed, if possible. It is evident, that all of Ann's sufferings are due to prolapsion of the womb, and the most natural course to be pursued to restore her health, to a physician who reasons abstractly, would be to replace the uterus, and retain it in its position by some mechanical means, such as a pessary. An instrument properly applied, and under proper circumstances, is often productive of much good; but in this case it could do no possible good, and perhaps lead to serious consequences. It is very plain, that the prolapsion is the effect of engorgement, which is the result of an inflammatory action about the os and cervix, for many years previous to the cessation of her catamenia. With these views the Lecturer advised the prolapsion to be let alone, and prescribed for the present, his favorite alterative tonic and laxative: ℞ Fraxinus Americana Cortex, ℥1, Asclepias Syriaca Radix, ℥ss, Brandy, Oi; one teaspoonful to be taken half an hour after each meal. And in addition to this Blue Mass, gr. iii, every third night.

In eight or ten days Ann reported herself very much better. Bowels tolerable regular, less headache, appetite gradually improved, strength increased. Advised the treatment to be continued.

